# Autoimmune Voltage-Gated Potassium Channel Limbic Encephalitis With Auditory and Visual Hallucinations

**DOI:** 10.7759/cureus.25186

**Published:** 2022-05-21

**Authors:** Bahadar S Srichawla

**Affiliations:** 1 Department of Neurology, UMass Chan Medical School, Worcester, USA

**Keywords:** paraneoplastic, cancer, hippocampus, psychiatry, potassium channel, voltage gated potassium channel autoimmune encephalitis, autoimmune limbic encephalitis, neuro immunology, clinical psychiatry, neurology

## Abstract

The limbic system (LS) coordinates an important role in memory generation, creating an emotional response to stress, and helping regulate autonomic and endocrine functions. Dysfunction of the limbic system can present secondary to many pathologies including autoimmune, infectious, paraneoplastic, etc. Lesions to the limbic system can also lead to varying symptoms which can be challenging for physicians to correctly identify and treat. Here we report a 59-year-old male with aggressive mood changes and acute onset of auditory and visual hallucinations. The cerebrospinal fluid (CSF) and serum immunological antibody panel confirmed the presence of voltage-gated potassium channel (VGKC) antibodies. Significant radiographic findings included an MRI revealing T2 hyperintensities in the bilateral hippocampus. Paraneoplastic screening with testicular ultrasound and chest CT was completed and was negative. A primary diagnosis of voltage-gated potassium channel limbic encephalitis (VGKC-LE) was made. Management included five days of intravenous immunoglobulin (IVIG) with subsequent resolution of symptoms. The limbic system is an intricate network of neurons that generates and relays key information to other parts of the brain. Its function and, in this case, its dysfunction remain an area of continued research. This case aimed to highlight the importance of recognizing the clinical presentation and objective findings of a rare type of autoimmune encephalitis and identifies the significance of paraneoplastic screening.

## Introduction

The limbic system consists of the cingulate gyrus, thalamus, hypothalamus, amygdala, and hippocampus. The hippocampus and amygdala execute the cardinal role of the limbic system. The coordination of these structures through complex neural circuits allows for the genesis of memories, and emotional response to stressors such as fear and anxiety [1]. Limbic encephalitis (LE) is known to have a varying clinical presentation that consists of spatial-temporal dissociation, severe anterograde amnesia, seizures, and mood disorder [2]. Most cases of limbic encephalitis are believed to be of paraneoplastic origin and only a few of pure autoimmune etiology. In fact, autoimmune limbic encephalitis has only been termed with current advances in the field of neuroimmunology and the discovery of new autoimmune antibodies [3]. Furthermore, the cases of voltage-gated potassium channel (VGKC) antibody-mediated autoimmune LE are sparse in the scientific literature [4]. Here, we reported a unique case of nonparaneoplastic autoimmune limbic encephalitis that occurred secondary to antibodies against voltage-gated potassium channels (VGKCs). The case relates the symptoms of auditory and visual hallucinations to the aforementioned neural circuits. We also emphasize the need for broad differential diagnoses and the importance of cancer screening in these patients.

## Case presentation

A 59-year-old man presented to the emergency department with subacute onset aggressive behavior and acute onset auditory and visual hallucinations. The patient was brought in by his daughter who noted her father had become increasingly aggressive during the past month. The patient endorsed that for the past day “demons” have been speaking to him and that he has visualized formed objects and people from his past who are not there. The patient denied any current usage of alcohol, tobacco, and recreational drugs. He endorsed a 20-pack-year history of smoking and quit approximately 15 years ago. The patient was alert and oriented to person, place, time, and situation. The physical examination was significant for constant irritability, agitated mood, and fixation on hallucinations. No focal neurologic deficits were noted on the examination. Vital signs were significant for a blood pressure of 107/72 mmHg, heart rate of 71 beats per minute, temperature of 37°C, and oxygen saturation by pulse oximetry of 98%.

A comprehensive metabolic profile (CMP) and complete blood count (CBC), vitamin B12, B9, and thyroid-stimulating hormone (TSH) levels were within normal limits. A comprehensive drug screen and an ethanol level were obtained which were negative. Cerebrospinal fluid (CSF) and serum studies were reviewed which were significant for elevated voltage-gated potassium channel (VGKC) antibodies at 124 pmol/L (normal <80 pmol/L) (Table 1). Notably, both leucine-rich glioma-inactivated protein 1 (LGI-1) and contactin-associated protein-like 2 (CASPR-2) antibody levels were negative.

**Table 1:** Cerebrospinal fluid (CSF) and serum immunological antibody panel. PCR: polymerase chain reaction

Lab parameters	Numerical values with reference range and units
Glucose, CSF	90 mg/dL (50-80)
Protein, CSF	42 mg/dL (15-45)
Red blood cell (RBC), CSF	3 (0)
Appearance, CSF	Clear
White blood cell (WBC), CSF	1
Venereal disease research laboratory (VDRL), CSF	Nonreactive
Lymphocytes %, CSF	40%
Mono/macrophage %, CSF	60%
Total cells counted, CSF	5
Immunoglobulin G (IgG), CSF	6.2 mg/dL (0.0-6.0)
Albumin, CSF	20 mg/dL (0-35)
Albumin nephelometry	2590 mg/dL (3500-5200)
Albumin index	7.7 (0.0-9.0)
Immunoglobulin G index	0.46 (0.28-0.66)
CSF IgG/albumin ratio	0.31 (0.09-0.25)
CSF oligoclonal bands	Negative
CSF IgG synthesis rate	<0.0
Herpes simplex 1 PCR, CSF	Nonreactive
Herpes simplex 2 PCR, SF	Nonreactive
*Haemophilus influenzae* DNA, CSF	Nonreactive
*Listeria monocytogenes* DNA, CSF	Nonreactive
*Streptococcus agalactiae* DNA, CSF	Nonreactive
*Streptococcus pneumonia* DNA, CSF	Nonreactive
Cytomegalovirus DNA, CSF	Nonreactive
Enterovirus DNA, CSF	Nonreactive
Human herpesvirus 6 DNA, CSF	Nonreactive
Human parechoviruses DNA, CSF	Nonreactive
*Cryptococcus neoformans*/*Cryptococcus gattii* DNA, CSF	Nonreactive
John Cunningham (JC) virus quantitative PCR, CSF	Nonreactive
Alpha-1 globulin	0.3 g/dL (0.2-0.3)
Alpha-2 globulin	0.7 g/dL (0.5-0.9)
Gamma globulin	1.5 g/dL (0.8-1.7)
Beta-1 globulin	0.5 g/dL (0.4-0.6)
Beta-2 globulin	0.3 g/dL (0.4-0.6)
Thyroid peroxidase antibody	1 IU/mL (<9)
Acetylcholine receptor ganglionic	<53 (<53)
Anti-SOX 1 Ab	<11 (<11)
AMPAR receptor (AMPAR) 1 Ab	Negative
AMPA receptor (AMPAR) 2 Ab	Negative
Antiphysin Ab	<11 (<11)
Type 1 antineuronal nuclear antibody (ANNA1) Ab	<11 (<11)
Type 2 antineuronal nuclear antibody (ANNA2) Ab	<11 (<11)
Type 2 antineuronal nuclear antibody (ANNA3) Ab	Negative
Aquaporin 4 Ab	Negative
Contactin-associated protein-like 2 (CASPR-2) Ab	Negative
CV2/collapsin response-mediator protein-5 (CRMP5) Ab	<11 (<11)
Dipeptidyl peptidase-like protein 6 (DPPX) receptor Ab	Negative
Epstein-Barr virus DNA, quantitative	Not detected
Gamma-aminobutyric acid-B (GABA-B) receptor Ab	<11 (<11)
Glutamic acid decarboxylase 65 (GAD65) Ab	<11 (<11)
Leucine-rich glioma-inactivated protein 1 (LGI-1) Ab	Negative
Ma2/Ta Ab	<11 (<11)
Myelin Ab	Negative
N-methyl-D-aspartate receptor (NMDAR) Ab	Negative
Purkinje cell cytoplasmic (PCA) Ab	Negative
Purkinje cell cytoplasmic type 1 (PCA1) Ab	<11 (<11)
Voltage-gated calcium channel (VGCC) type N Ab	<55 (<55)
Voltage-gated potassium channel (VGKC) protein complex, Ab	112 pmol/L (<80)
Voltage-gated calcium channel (VGCC) protein complex Ab	<30 pmol/L (<30)
Zinc protein 4 (Zic4) Ab	<11 (<11)

**Figure 1:**
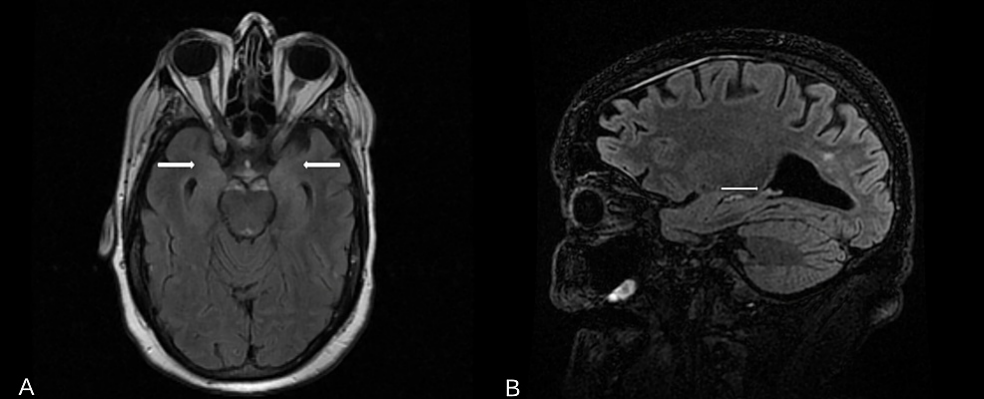
MRI brain with and without contrast T2/FLAIR images. (A) MRI T2/FLAIR axial view and (B) MRI T2/FLAIR sagittal view. FLAIR: fluid attenuated inversion recovery

Further microbiologic testing was completed including hepatitis C virus (HCV) polymerase chain reaction (PCR), cytomegalovirus (CMV) DNA PCR, quantitative HCV RNA, hepatitis B antibody and antigen, HIV Ag/Ab (fourth generation), and SARS-CoV-2 RNA PCR all showed nonreactivity. An MRI brain with and without contrast was obtained for further evaluation, which revealed mild T2 hyperintensities of the bilateral hippocampus (Figures 1A, 1B). Based on the clinical presentation and diagnostic data obtained, a diagnosis of voltage-gated potassium channel limbic encephalitis (VGKC-LE) was made.

Given the known correlation between limbic encephalitis and testicular malignancies, a bedside ultrasound of the scrotum was completed. The ultrasound did not reveal any malignant lesions, only bilateral benign cysts of the testicular heads (Video 1). A high-resolution computerized tomography (CT) of the chest was also completed revealing no signs of malignancy. The patient was subsequently started on five days of intravenous immunoglobulin (IVIG) for treatment. On the fourth day of IVIG therapy, all visual and auditory hallucinations had ceased, and the patient was noted to have a calm mood. The patient was subsequently discharged after completing five days of IVIG therapy.

**Video 1:** Testicular ultrasound revealing no malignant lesions, cyst on the right testicular head.

At a six-month neurology follow-up, the patient accepts no visual or auditory hallucinations and no changes in mood and affect. Approximately 1.5 years after the initial presentation the patient once again complained of acute onset auditory and visual hallucinations. Repeat serum antibody studies at that time showed elevated VGKC protein complex antibodies at 126 pmol/L (normal <80 pmol/L). Treatment was started with IVIG for five days, which led to the resolution of symptoms. The patient since then was discharged and remains symptom-free.

## Discussion

Limbic encephalitis occurs most often due to paraneoplastic origin, however, infectious and autoimmune etiologies are possible. Recent studies have shown the prevalence of both infectious and autoimmune LE are comparable; however, the incidence of autoimmune LE is increasing [5]. Within high-income countries, it is estimated that LE affects five to 10 of 100,000 residents per year [6]. The increasing incidence of autoimmune encephalitis is likely due to improved diagnostic testing with a wider array of autoimmune antibodies being identified including N-methyl D-aspartate receptor (NMDAR) and in our case voltage-gated potassium channel (VGKC) autoantibodies. Anti-NMDAR-mediated encephalitis is still the most common form of autoimmune encephalitis identified in the literature. It is estimated to make up more than 20% of all cases identified [7]. Infectious etiologies have also been recognized in creating inflammation of the limbic system; common offenders include the West Nile virus (WNV), which is known to primarily affect subcortical structures as well as John Cunningham virus (JCV), human herpesvirus (HHV) 6 and varicella-zoster virus (VZV) [5]. Autoimmune encephalitides are known to have varying gender frequencies based on their subtypes; however, most cases occur in women. Most notably the contactin-associated protein-like 2 (CASPR-2) mediated encephalitis is known to have a skewed female-to-male ratio of 1:9 and also often presents in older adults [8].

VGKC mediated encephalitides are described in three distinct phenotypes in adults. Leucine-rich glioma-inactivated protein 1 (LGI-1), contactin-associated protein-like 2 (CASPR-2), or seronegative subtype. Both LGI-1 and CASPR-2 are found within the VGKC protein complex [8,9]. In our case, both LGI-1 and CASPR-2 antibodies were found to be negative; however, VGKC protein complex antibody levels were elevated. Thus, the symptoms present and underlying encephalitis seen in this case are likely due to another antigenic compound within the VGKC protein complex which is yet to be characterized.

Psychiatric manifestations of limbic encephalitis are not uncommon and can include personality changes, aggressive behavior, and hallucinations, as in our case. These symptoms can mistakenly be characterized as an acute psychotic episode or schizophrenia [7]. Many of these psychiatric symptoms can be associated with focal neurologic deficits as well, and thus it is prudent to perform a comprehensive neurologic examination on these patients. If focal deficits are observed, further neurologic evaluation is warranted by neuroimaging and/or spinal tap. In fact, it has previously been reported that up to half of the patients with new-onset psychiatric symptoms with concurrent neurologic findings on examination never undergo further neurodiagnostic tests [10]. However, in our case, the patient presented psychiatric manifestations without focal neurologic deficits on the physical examination. Given the low suspicion of new-onset psychosis or schizophrenia at the age of 59 years, a neurologic cause was immediately part of the differential. This is further supported by the patient’s smoking history of 20-pack-year and concern for a paraneoplastic process.

**Figure 2:**
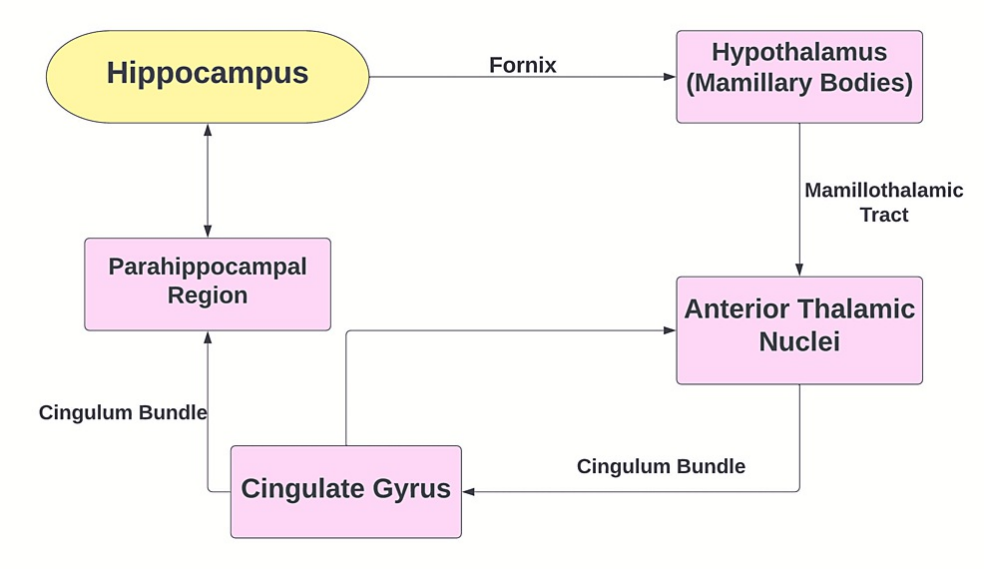
The circuit of Papez is a neural loop that helps regulate emotional expression.

Hallucinations can occur in all sensory types including visual, auditory, and tactile. The hippocampus is a major part of the limbic system, and here lesions are hypothesized to play a role in hallucinations seen in many neuropsychiatric conditions. The hippocampus receives input from the entorhinal cortex (EC) and is relayed through the sub-hippocampal fields (CA1-4). This information is then relayed to the EC and to other cortical and subcortical structures, including the prefrontal cortex, mamillary bodies, and thalamus, as well as other limbic structures (Figure 2) [11]. Lesions in these hippocampal structures as seen in our patient have been associated with predominantly visual hallucinations and less correlated with auditory hallucinations [12,13]. Similarly, in our case, we appreciate mild T2 hyperintensities within the bilateral hippocampus which play a contributory role in the auditory and visual hallucinations witnessed. Most cases of symptomatic VGKC limbic encephalitis have subcortical lesions often involving the limbic system. Biomarkers of axonal damage such as neurofilament light (NF-L) are also found to be significantly elevated in autoimmune encephalitis in comparison to coronavirus disease of 2019 (COVID-19) encephalopathy [14].

The diagnosis of limbic encephalitis remains to be of growing incidence, as previously mentioned, occurring primarily through expanding CSF and serum antibody panels and through radiographic findings that include lesions in the limbic system and, most notably, the hippocampus. Seronegative limbic encephalitides continue to be challenging for physicians to diagnose and further manage. Many such cases require multidisciplinary support from neurologists and psychiatrists to be adequately managed [15]. Management of LE occurs primarily through treatment of the underlying origin. Paraneoplastic LE is best managed through treatment of the underlying malignancy, and infectious LE through eradicating the causative agent. The management of autoimmune LE remains to be high-dose steroids and/or intravenous immunoglobulin (IVIG). Most cases of autoimmune LE have been noted to have a favorable response to a five-day treatment course of IVIG; however, few refractory cases have been noted requiring therapeutic plasma exchange (TPE) [16]. In our case, IVIG therapy led to favorable outcomes and the patient remained disease-free for 1.5 years until symptoms returned and resolved once again with a five-day IVIG course.

Voltage-gated potassium channel autoantibody-mediated limbic encephalitis remains to be a rare form of LE. Its clinical symptoms are variable; however, most cases have been found to have significant psychiatric overlap [17]. There are currently no clinical guidelines on cancer screening for these patients. Most forms of paraneoplastic limbic encephalitis occur secondary to lung, testicular, and breast cancer [18]. Given the high correlation of limbic encephalitis to a paraneoplastic origin screening of lung and testicular cancer is warranted in males. And similarly screening for lung and breast cancer in females is also needed. Serial imaging and cancer screening are important in these patients as they may show radiographic evidence of malignancy in the future. Suspicion of lung cancer and the need for screening with new LE is also justified with a history of current or previous tobacco usage as seen in this case. Further research, on the exact pathophysiology of VGKC in the hippocampus and associated limbic structures, is needed to correlate with the psychiatric symptoms described.

## Conclusions

The limbic system is a complex network of neurons that aid in governing the creation of new memories, defining key traits of our personality, and generating a response to emotional stressors. Voltage-gated potassium channel limbic encephalitis (VGKC-LE) remains to be a rare form of autoimmune encephalitis that is still not well characterized in terms of symptoms, diagnostics, or treatment in scientific literature. Here we reported a case of VGKC-LE that was seronegative for both LGI-1 and CASPR-2 antibodies that presented with auditory and visual hallucinations. Treatment strategies including intravenous immunoglobulin, plasma exchange, and steroids are discussed. The importance of cancer screening (lung, breast, and testis) in these patients is described. Further identification of antigenic compounds within the VGKC protein complex is needed.
